# Development of an anti-rat complement C2 antibody that improves renal outcome in a rat kidney transplant model

**DOI:** 10.3389/fimmu.2025.1668376

**Published:** 2026-01-15

**Authors:** Laura Bracke, Jolien Delaere, Eline Haspeslagh, Karen De Winter, Yasmine Driege, Raphael Bilgraer, Tim Delahaye, C. Erik Hack, Inge Van de Walle

**Affiliations:** argenx, Ghent, Belgium

**Keywords:** complement C2, rat model, ischemia-reperfusion, delayed graft function, antibody, transplant, kidney

## Abstract

**Background:**

Previously we reported on the therapeutic monoclonal anti-human C2 antibody empasiprubart that inhibits activation of the classical and lectin pathways of complement. Preclinical studies with this antibody are hampered by its low affinity for C2 of animal species other than primates.

**Methods and results:**

We developed a high affinity, Ca^2+^-dependent anti-rat C2 antibody using the sequences and structural data of empasiprubart. Pharmacokinetics and pharmacodynamics of the resulting antibody in Sprague Dawley rats were assessed and used for an intervention study in a rat model of delayed graft function following kidney transplantation. The anti-rat C2 antibody improved kidney function and health in the rats within the first 2 weeks post-transplantation.

**Conclusion:**

Our study shows the successful development of an analogue of empasiprubart that can be used in preclinical *in vivo* disease models and highlights the potential of C2-blocking as a therapeutic strategy for preventing delayed graft function following kidney transplantation.

## 1 Introduction

**Figure 1:**
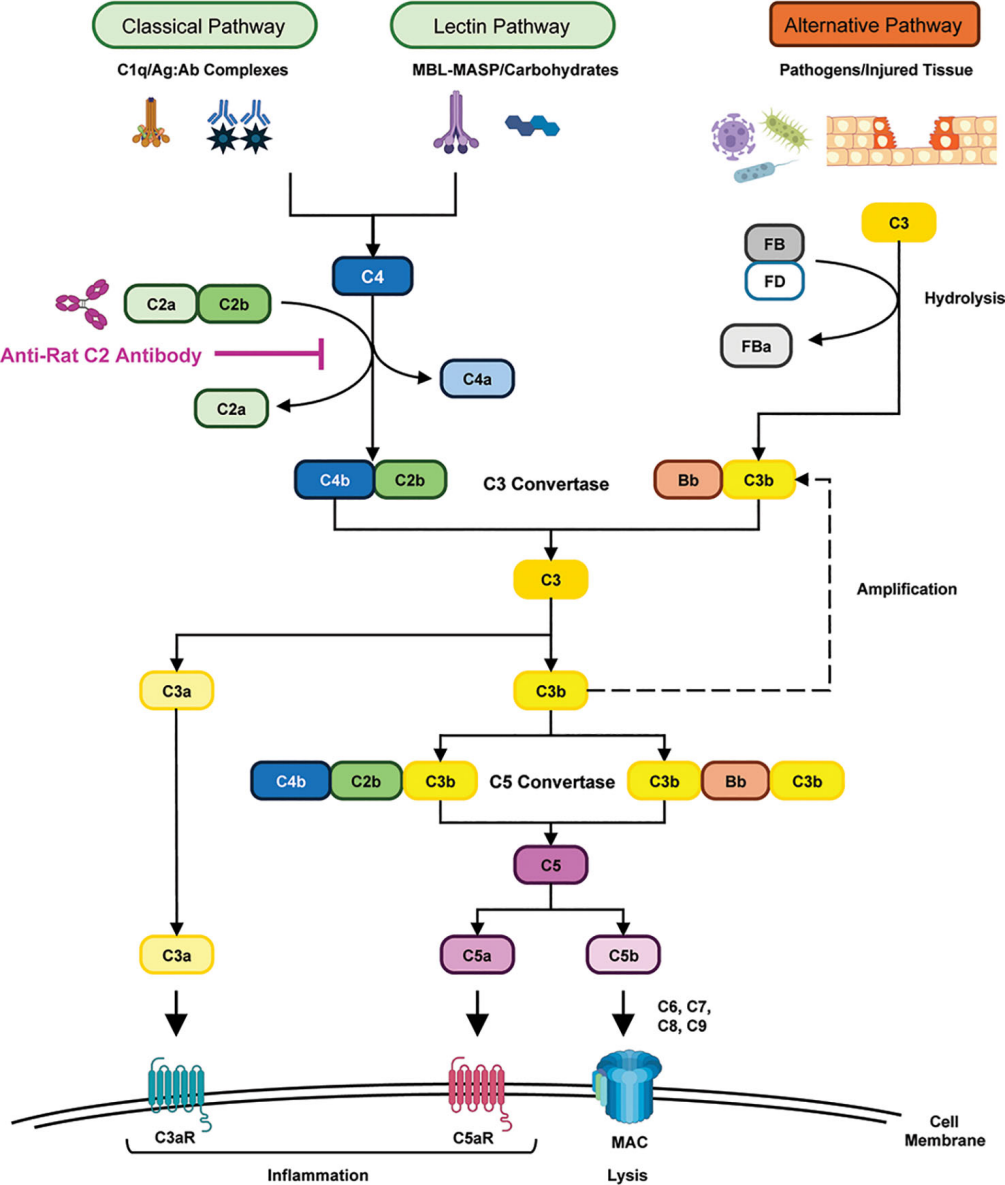
The complement cascade. The complement system, consisting of more than 30 proteins, can be activated via three pathways: the classical pathway (CP, triggered by e.g. antigen-antibody complex formation), the lectin pathway (LP, triggered by e.g. carbohydrate recognition), and the alternative pathway (AP, triggered e.g. by complement factor deposition on pathogen surfaces or damaged tissue). The CP and LP converge at the level of C4 activation. CP/LP further converges with the AP at the level of C3 convertase formation, which cleaves C3 into C3a (an inflammatory mediator) and C3b (an opsonin). C3b additionally binds C3 convertases to form C5 convertases, which cleave C5 into C5a (another inflammatory mediator) and C5b, thereby initiating assembly of the Membrane Attack Complex (MAC). The MAC forms pores in the target cell membrane, resulting in cell lysis and death of the targeted cell. Regulatory proteins prevent excessive immune activation and tissue damage (1–3). Binding of empasiprubart/anti-rat C2 antibody to the C2a part of C2 blocks the formation of the C4bC2 complex and hence blocks the activation of CP and LP upstream of C3. Figure adapted from Van de Walle I, et al. *Nat Commun* 2025;16:7639 (10) under a CC BY-NC-ND 4.0 license. MBL, mannose binding lectin; ag:ab, antigen:antibody; MASP, mannan-binding lectin-associated serine protease; C3aR, C3a receptor; C5aR, C5a receptor.

The complement system is part of the innate immune system and as such contributes to the defense against invading pathogens, removal of cellular debris, modulation of adaptive immune system responses, and maintenance of the body’s homeostasis. The system consists of more than 30 proteins, which can be activated via three pathways, the classical (CP), lectin (LP), and alternative (AP) pathways, that converge at the level of the third complement component, C3, to activate a final common pathway leading to the formation of membrane attack complexes (MAC) (1–3). During activation, several proinflammatory peptides and protein complexes are generated such as C3a, C5a, and MAC (**Figure 1**).

The proinflammatory potential of complement is important for its physiological function, but when not properly controlled, can kill host cells, and contribute to inflammatory tissue damage. Indeed, complement dysregulation drives pathology in many human diseases including age-related macular degeneration (4), auto-antibody-mediated diseases such as myasthenia gravis (5), and ischemia-reperfusion (IR) conditions like stroke or delayed graft function (DGF) after kidney transplantation (6, 7).

To attenuate pathologic complement activation in human disease, various inhibitors have been developed. Among approved inhibitors for clinical application are the anti-C5 antibodies eculizumab and ravulizumab, plasma-derived and recombinant C1 inhibitors, the C5aR1 antagonist avacopan, the factor B inhibitor iptacopan, and sutimlimab, a C1s-specific antibody (8). This variety of complement inhibitors reflects the complexity of the complement system and its biological effects, and their varying benefit-to-risk ratio depending on the clinical condition and underscores that there is no “one-size-fits-all” inhibitor to modulate complement in human disease. Therefore, other complement factors are being evaluated as targets for therapeutic intervention. Among these is C2, which participates in CP and LP, but not in AP, which plays a major role in anti-bacterial defense. Previously, we reported on empasiprubart (formerly ARGX-117), a humanized recycling monoclonal antibody (mAb) that binds to C2 in a pH- and Ca^2+^ -dependent manner (9). Binding of empasiprubart blocks the formation of the C4bC2 complex by binding to the C2a part of C2 (following recommended nomenclature in this paper the term C2a is used to indicate the smaller subunit of C2) and hence blocks the activation of CP and LP upstream of C3 (**Figure 1**). A first-in-human study shows that empasiprubart is safe and well-tolerated and has long-lasting pharmacodynamic effects (10).

A potential indication for anti-C2 treatment is IR injury (IRI), which among others occurs in DGF following kidney transplantation. Evaluation of empasiprubart in preclinical models of DGF and other disease conditions, however, is hindered by its limited cross-reactivity with C2 of animal species other than primates. The anti-human C2 antibody empasiprubart was previously shown to bind with a 30-fold lower affinity to rat C2 (*K*_D_ = 9.08 nM) than to human C2 (*K*_D_ = 0.3 nM), whereas it did not bind to mouse, guinea pig, or rabbit C2 (9). This study describes the generation and characterization of a high affinity Ca^2+^-dependent monoclonal antibody targeting C2 of rats, cynomolgus monkeys, hamsters, and humans, including its pharmacokinetic (PK) and pharmacodynamic (PD) properties in rats. To evaluate the *in vivo* efficacy of the new anti-rat C2 antibody and demonstrate its potential as a tool for proof-of-concept studies in animal models, the antibody was tested in a rat kidney transplantation model, in which it was previously shown that pharmacological inhibition of complement during IR prevented immediate graft loss (11).

## 2 Material and methods

### 2.1 Development of the anti-rat C2 antibody

Synthetic genes (VH and VL of empasiprubart) were generated by PCR-based assembly of overlapping oligonucleotides with random mutations on selected positions in the CDR regions based on empasiprubart’s crystal structure (12). After gene assembly via PCR, amplicons were digested and ligated into the phagemid vector as previously described (13). Phages were produced as described by van der Woning et al. (14) and screened for rat C2 binding.

### 2.2 Generation and production of antibodies for *in vitro* analyses

All antibodies for *in vitro* analysis were produced as hIgG1 with L234A/L235A (LALA) mutations following protocols described elsewhere (15). Briefly, HEK293 cells were transfected with 1:1 heavy/light chain plasmid ratio using 1:3 DNA/PEI ratio for transient production followed by protein A affinity purification.

### 2.3 Generation and production of antibodies for *in vivo* analyses

HEK293E-253 cells were transfected with endotoxin-free plasmid DNA using the rPEx^®^ Technology at ImmunoPrecise Antibodies Ltd (the Netherlands) to produce antibodies for *in vivo* analysis. Six days post-transfection conditioned medium containing recombinant antibody was harvested using SatroClear Dynamics, followed by MabSelect PrismA purification. Obtained antibodies were sterilized by filtration over a 0.22 µm syringe filter.

### 2.4 Rat CP and LP assay

Microtiter wells (Greiner Bio-one, Cat N°: 675061) were coated with immunoglobulin (Ig)G (CP) or mannan (LP) and incubated overnight at 4 °C or at room temperature, respectively. After blocking with 1% w/v casein (Bio-Rad Laboratories, Cat N°: 1610783) and washing, wells were incubated with rat serum samples diluted in veronal buffered saline at 37 °C. As controls, MgEGTA (inhibits CP and LP) or EDTA (inhibits all pathways) were added to the buffer. After washing, wells were incubated with biotinylated anti-rat C3 antibody (Abcam, Cat N°: ab17456), followed by streptavidin-HRP (Pharmingen, Cat N°: 554066). Afterwards, wells were washed and incubated with TMB solution. The substrate reaction was stopped with sulfuric acid and OD450/620 nm was measured with a spectrophotometer (Infinite M Nano, Tecan).

### 2.5 Rat AP assay

Rat AP activation levels were assessed using a commercially available ELISA kit (Hycult Biotech, HIT412, Uden, the Netherlands). Serum was diluted to 2% v/v with kit provided sample dilution buffer. The manufacturer’s instructions were followed, and absorbance was measured at 450 nm. In addition to the kit’s positive and negative control, healthy pooled rat serum was added as positive control (InnoSer, Diepenbeek, Belgium).

### 2.6 Cross-species C2 binding ELISA

Briefly, wells of a MaxiSorp 96-well plate (ThermoScientific, Cat N°: 442404) were coated with human, cynomolgus monkey, guinea pig, rabbit, mouse, hamster, or rat recombinant C2 protein (produced in HEK293E-253 cells, ImmunoPrecise Antibodies Ltd, the Netherlands) overnight at 4 °C, blocked with 1% w/v casein and incubated with 5 µg/mL of the anti-rat C2 antibody or empasiprubart. After washing, wells were incubated with HRP-conjugated goat anti-human IgG antibodies (Bethyl Laboratories, Cat N°: A80-319P). Afterwards, wells were washed and incubated with TMB solution. The substrate reaction was stopped with sulfuric acid and OD450/620 nm was measured with a spectrophotometer.

### 2.7 Meso Scale Discovery experiments

Multi-array 96-well plates (Meso Scale Discovery, Cat N°: L15XA-3) were coated overnight at 4 °C with recombinant rat C2. After washing the plates with wash buffer A (**Supplementary Table 1A**), wells were blocked with 1% w/v Bovine Serum Albumin (BSA) (Sigma-Aldrich, Cat N°: 05482). Subsequently, plates were washed with appropriate wash buffer (**A-D, Supplementary Table 1A**) and incubated with serial dilutions of antibody in the corresponding dilution buffer (**A-D, Supplementary Table 1A**). After washing the plates with appropriate wash buffer (**A-D, Supplementary Table 1A**), a final wash step with buffer A was performed. Next, Meso Scale Discovery-Sulfo tag-labeled goat anti-human IgG antibody (Meso Scale Discovery, Cat N°: R32AJ-1) was applied. Finally, wells were washed, MSD reading buffer (Meso Scale Discovery, Cat N°: R92TC-2, diluted ½ in MQ water before use) was added to the wells and signal was measured immediately by Meso™ QuickPlex SQ 120 (Meso Scale Discovery).

### 2.8 C2 affinity determination via SPR

The surface plasmon resonance (SPR) measurements were performed on a Biacore T200 instrument (Cytiva). Recombinant rat C2 was diluted to 5 µg/mL in immobilization buffer (10 mM sodium acetate pH 4.5, Cytiva, Cat N°: BR100350), injected and immobilized to 150 response units on the surface of a carboxymethylated dextran sensor chip (Sensor Chip CM5 series S, Cytiva, Cat N°: 29104988) at a flow rate of 30 µL/min. Binding measurements of empasiprubart and the anti-rat C2 antibody were performed in 20 mM HEPES, 100 mM NaCl_2_, 1.25 mM CaCl_2_, 0.05% Tween20, pH 7.5, at 30 µL/min flow rate. After each measurement, the surface was regenerated by two injections of 10 mM glycine-HCl pH 1.5 (Cytiva, Cat N°: BR100354) at a flow rate of 30 µL/min without loss of C2 binding capacity. Sensorgrams were generated at concentrations of 33.33, 16.67, 8.33, 4.17, 2.08, 1.04, and 0.52 nM antibody. Kinetics of the antibodies were determined with the BIAevaluation software (version 5.0.18) using simultaneous *k*_a_/*k*_d_ fitting with 1:1 mass transfer.

### 2.9 ELISA for free rat C2

MaxiSorp microtiter plates were coated overnight with the anti-rat C2 antibody and blocked with 3% w/v BSA. After blocking, samples and calibration curve were pipetted into the ELISA plates and incubated on ice. Serum samples were diluted in assay buffer (TBS: Tris Buffered Saline, ThermoFisher, Cat N°: BP24711) supplemented with 2 mM CaCl_2_ (Sigma, Cat N°: 21115). As a calibration curve, dilutions of recombinant rat C2 in assay buffer were used. After incubation and washing, free C2 was detected with biotinylated human anti-rat C2 antibody 3H07 (argenx). The epitope targeted by 3H07 is distinct from that recognized by the newly developed anti-rat C2 antibody. After incubation on ice, plates were developed with streptavidin-HRP and s(HS)TMB (SDT reagents, Cat N°: sTMB) ½ diluted in MilliQ. OD450/620 nm was measured with a spectrophotometer after the reaction was stopped with sulfuric acid.

### 2.10 ELISA for total rat C2

High-binding half-area plates were coated overnight with mouse anti-rat C2 antibody 3H07. The following day, plates were blocked using 1% v/v casein in PBS and washed. Next, samples and calibration curve diluted in TBS supplemented with 2 mM CaCl_2_, 10 µg/mL of the new anti-rat C2 antibody and 10%, v/v rabbit serum (Cederlane, Cat N°: CL3441S50), were incubated. The calibration curve consisted of dilutions of recombinant rat C2 in assay buffer. After incubation and washing, plates were developed with goat anti-human IgG Fc HRP (Abcam, Cat N°: ab97225) and subsequently TMB.

### 2.11 Rat PK assay

In brief, Maxisorp ELISA plates were coated with mouse anti-hIgG (CH2) (Bio-Rad Laboratories, Cat N°: 18570060) on which serum samples containing the anti-rat C2 antibody, 1/100 diluted in 1x PBS/0.1% v/v casein, were incubated. After a wash step, plates were incubated with HRP-conjugated goat anti-human IgG antibodies (Bethyl Laboratories, Cat N°: A80-319P). After a final wash step, plates were developed using TMB. Each run contained a calibrator curve (11 non-blank known anti-rat C2 antibody concentrations spiked in rat serum) and at least two sets of quality controls. Concentrations of the anti-rat C2 antibody in the study samples and QC samples were calculated from the calibrator curve using a five-parameter logistic (5PL) fit without additional weighting factors applied.

### 2.12 Rat PK/PD study

Eight-week-old male Sprague Dawley rats weighing 250–300 g were used for a PK and PD study of the anti-rat C2 antibody. Animals were housed under conventional conditions at InnoSer (Diepenbeek, Belgium). A single dose of 2, 5, 10, 25, or 50 mg/kg anti-rat C2 antibody (hIgG1 LALA) was administered intravenously (IV). Each dose group included three animals. As a control group, 3 rats received 50 mg/kg isotype control (Mota hlgG1 LALA). Baseline blood samples were collected 7 days and 1 day prior to the study’s start. Intermediate blood samples were taken at 5 minutes, 6 hours, 24 hours, 48 hours, 3 days, 5 days, 7 days, 14 days, and 21 days post-dosing without anesthesia. After the final tail vein blood collection on day 21, all animals were euthanized humanely via an intraperitoneal overdose of Euthasol vet (200 mg/kg). For free C2, the relative percentage change of concentration, normalized to the mean baseline value within each group, was determined. A non-compartmental analysis was used for parameter estimation using Phoenix WinNonlin (Certara LP, USA). The IV bolus model (Calculation method: Linear Up, Log Down) was used on individual time-concentration curves, using nominal dose levels and nominal sampling times relative to the dose administration.

Population PK/PD modeling was performed using NONMEM version 7.5.1 (ICON Development Solutions, Ellicott City, MD, USA), employing the stochastic approximation expectation-maximization algorithm for parameter estimation. Due to a high proportion of PD data below the limit of quantification (BLQ), the M3 method was applied to incorporate BLQ data into the likelihood calculation and ensure robust parameter estimation. Diagnostic plots and visual predictive checks were used to evaluate model performance. Simulations based on the final population PK/PD model were conducted in a virtual rat population (n = 1000) to determine an optimal dosing regimen of the anti-rat C2 antibody that maintained complete suppression of free C2 levels over a 7-day period.

### 2.13 Rat orthotopic kidney transplant model

The study was performed in the Charles River facility in Laval (Quebec, Canada) according to internal standard operating procedures and approved by the local animal ethical committee (Study 20441571, IACUC CRL Montreal ULC-Laval). Syngeneic inbred, male, adult Lewis rats (CRL, North Carolina, USA) weighing 300–400 g served as donors and recipients to avoid graft rejection. The animals were housed under conventional conditions. Kidney transplantation was performed as adapted from Yu et al. (11). The left donor kidney was flushed with heparinized saline (80 U/mL) followed by a flush with cold preservation solution (Belzer UW cold storage solution) before removal. The kidney was then placed in a sterile container with UW cold storage solution for 32 hours at 4 °C and then kept on ice pending transplantation. After left nephrectomy of the recipient (10–11 weeks), the donor kidney was transplanted by connecting the arterial and venal cuff to the recipient abdominal aorta and vena cava, respectively. Next, the ureter was connected by end-to-end anastomosis, and the kidney was reperfused. After the left kidney transplant, the recipient right kidney was removed and discarded. Isoflurane at 2–3% was used to anesthetize the rats during the procedure. Animals were sacrificed via exsanguination from the abdominal aorta after isoflurane anesthesia, unless deemed inappropriate by the Study Director and/or the clinical veterinarian, when humane endpoints were reached following CRL SOPs. Surgery day was defined as day 1.

Recipient animals were dosed IV in the tail vein immediately prior to reperfusion with a bolus of 50 mg/kg anti-rat C2 antibody (18 animals) or a hIgG1 LALA isotype control mAb (9 animals), followed by a second intravenous bolus injection of 25 mg/kg 72 hours post-transplant. Animal mortality within 24 hours post-surgery was considered related to surgical procedure, and these animals were replaced. Blood samples were collected on day 3, 5, 7, and 14 for assessment of renal function (serum creatinine and blood urea nitrogen [BUN] levels) and biomarker (Neutrophil Gelatinase-Associated Lipocalin (NGAL)) analysis. On day 14, animals were sacrificed for histological examination and weighing of the transplanted kidney. Due to a few unscheduled deaths at earlier time points (n = 3 per group), and 7 prospective scheduled deaths for early kidney histology (data not shown), final sample sizes were: 18, 11, 9, and 7 for the anti-rat C2 mAb group and 9, 8, 7, and 6 for the isotype control mAb group on days 3, 5, 7, and 14 respectively.

### 2.14 Blood and histology samples

Rat blood samples were collected in normal Eppendorf tubes and clotted for 30 min at room temperature followed by centrifugation (4 °C, 1300 - 2000 × g, 10 min). Serum samples were aliquoted and snap-frozen within 2 hours after sampling. Samples were stored at -70 °C and were not subjected to freeze-thaw cycles prior to analysis of CP, free C2, total C2, and PK. Unfrozen serum samples were analyzed for BUN by *in vitro* kinetic testing with urease and glutamate dehydrogenase, and for creatinine by the Jaffé alkaline picrate method, both on a Roche/Hitachi Cobas c analyzer according to the packaging inserts (Ref ID 04460715 190 and 4810716 190, respectively). Transplanted kidneys were dissected, weighted, and then stored in 10% neutral buffered formalin, followed by paraffin embedding, sectioning, mounting, and staining with hematoxylin and eosin for microscopic analysis.

### 2.15 Statistical analysis

Log-transformed serum creatinine and BUN concentrations at day 3 were analyzed with a linear model including treatment group as a fixed effect and a heterogeneous residual variance across treatment groups. A longitudinal linear mixed model was fitted to the log-transformed concentrations of serum creatinine, BUN and normalized NGAL (= fold increases to healthy serum) obtained at days 3, 5, 7, and 14. Treatment group, day and their interaction were included as categorical fixed effects. A flexible correlation structure heterogeneous across treatment groups was incorporated to correlate the outcomes between consecutive time points within one animal: heterogeneous compound symmetry for serum creatinine, and heterogeneous first-order autoregressive structures were applied for BUN and normalized NGAL. The linear models were fitted using restricted maximum likelihood and the Kenward-Roger approximation for the degrees of freedom using SAS v9.4. Wald hypothesis tests were used to compare the (time profiles of) log-transformed concentrations of serum creatinine, BUN, and normalized NGAL between treatments. Results were visualized using GraphPad Prism v10. *p*-values <0.05 were considered statistically significant.

## 3 Results

### 3.1 Generation and characterization of the anti-rat C2 antibody

**Figure 2:**
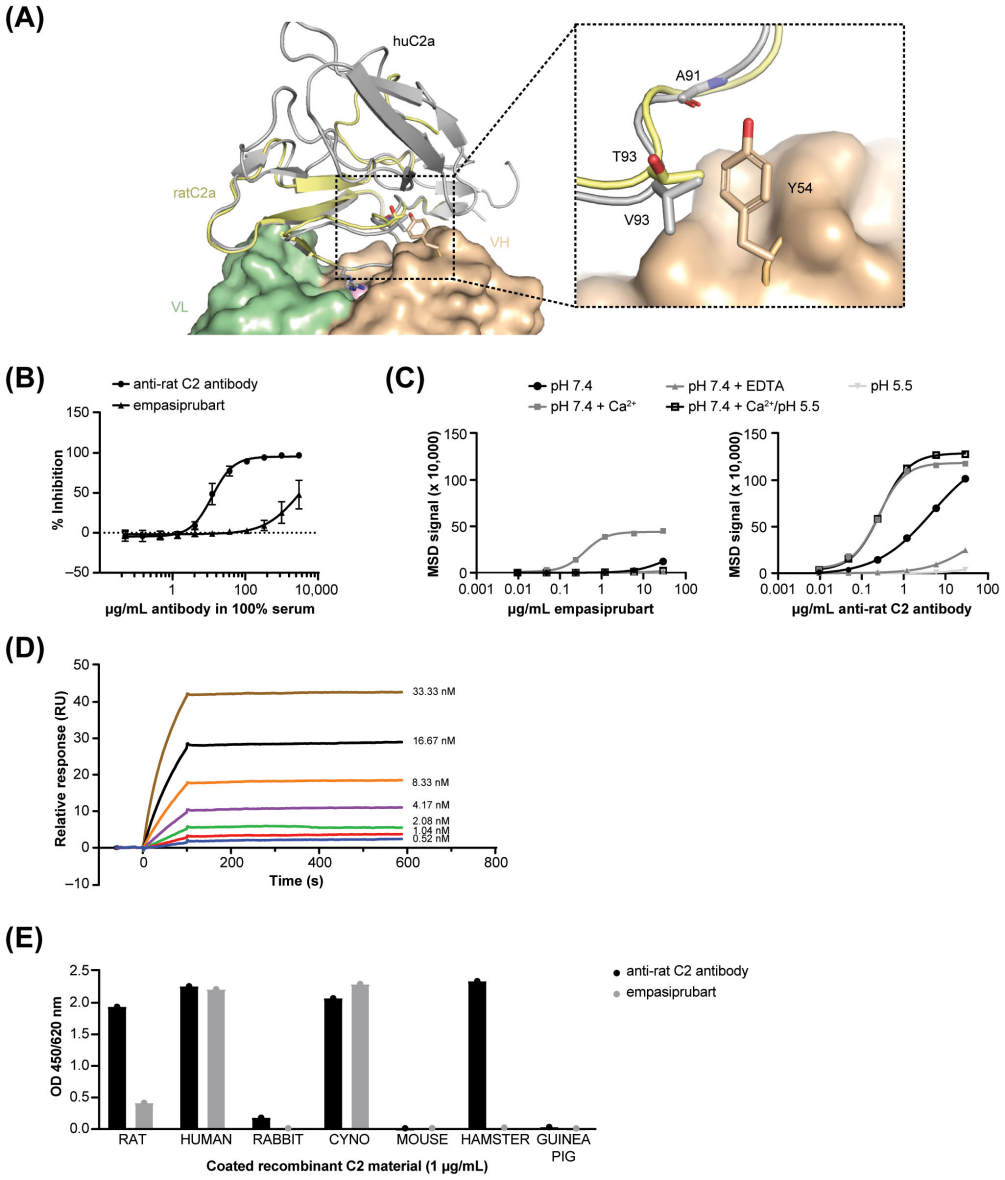
Generation and characterization of a rat anti-C2 blocking antibody. **(A)** Cartoon representation of the empasiprubart Fab-C2a complex in the presence of calcium ion (pink sphere) with superimposition of a rat C2a model. Empasiprubart VH is shown in orange, empasiprubart VL in green, human C2a in gray and a model of rat C2a in yellow. **(B)** The potencies of empasiprubart and anti-rat C2 antibody to inhibit activation of CP of rat complement. Pooled rat serum, pre-incubated with the indicated antibody, was incubated with solid-phase IgG. C3 deposition was then assayed. **(C)** Binding of empasiprubart or anti-rat C2 antibody to rat C2 under different conditions as assessed with MSD. Binding and washing steps were both performed with the indicated condition (pH 7.4, pH 5.5, pH 7.4 + 1.25 mM Ca^2+^, and pH 7.4 + 20 mM EDTA) except for the pH 7.4/pH 5.5 condition of which binding was performed at pH 7.4 and washing at pH 5.5 to mimic the *in vivo* endocytosis. **(D)** Kinetic binding profiles of anti-rat C2 antibody at 0.52 nM–33.33 nM to rat C2 as analyzed by SPR at pH 7.4. **(E)** Binding of the anti-rat C2 antibody or empasiprubart (5 µg/mL) to solid-phase recombinant C2 of different species at pH 7.4.

Superimposition of a predicted model of rat C2a (AF-A0A0U1RRP9-F1) on top of our previously published crystal structure of empasiprubart bound to CCP2 of human C2 (PDB 8ACI, manuscript submitted) suggested that the weak affinity to rat C2 was possibly due to the presence of a threonine in rat C2 at the equivalent position of V93 in human C2 (10). The more polar side chain of threonine likely stabilizes the position of the side chain of HCDR3 Y54 which interacts less efficiently with the main chain of C2 A91 (**Figure 2A**).

Using CDR randomization and phage display techniques, several candidate Fabs derived from empasiprubart’s sequence showed increased binding to rat C2 and were produced as human IgG1 antibody for further characterization (data not shown). *In vitro* analysis of these mAbs yielded one particular mAb that stood out as an inhibitor of both CP (IC_50_ of 7.45 µg/mL, **Figure 2B**) and LP (IC_50_ of 16.10 µg/mL, **Supplementary Figure 1A**) activity without interfering with AP activity of rat complement (**Supplementary Figure 1B**). This anti-rat C2 antibody demonstrated increased binding to rat C2 as compared to empasiprubart (maximum MSD signal of 101.58×10^3^ and 12.04×10^3^ at pH 7.4, respectively) (**Figure 2C**). Moreover, binding of the antibody to rat C2 was also calcium-dependent like empasiprubart to human C2, as illustrated by the increase and decrease in maximum MSD signal at neutral pH with addition of calcium or EDTA, respectively, to the buffer (117.85×10^3^ vs 24.66×10^3^ and 45.04×10^3^ vs 1.07×10^3^, **Figure 2C**). Lowering the pH from 7.4 to 5.5 impaired the binding capacity of the newly developed antibody to rat C2 (maximum MSD signal 3.79×10^3^ vs 0.32×10^3^). However, once bound to C2 at neutral pH (with calcium), a lower pH did not disrupt the binding as indicated by the overlapping curves. Consequently, the anti-rat C2 antibody, in contrast to empasiprubart, lacks pH-dependent target release properties for rat C2. Finally, affinity data confirmed low cross-reactivity of empasiprubart to recombinant rat C2 (**Supplementary Figure 1C**) whereas the anti-rat C2 antibody binds to both recombinant rat C2 (**Figure 2D**) and recombinant human C2 with high affinity at pH 7.4 (**Supplementary Figure 1C**). No *k*_d_ values could be calculated due to the low off-rate of the anti-rat C2 antibody. Finally, cross-reactivity of the anti-rat C2 antibody to other species was assessed (**Figure 2E**). Next to strong C2 binding for rat, the antibody illustrated cross-reactivity to human, cynomolgus monkey, and hamster C2. To reduce unwanted stimulation of effector functions in rats, the anti-rat C2 antibody was formatted as a hIgG1 with L234A/L235A (LALA) mutations, diminishing its interaction with Fc gamma receptors and C1q (16).

Thus, an anti-rat C2 antibody was developed that exhibits a Ca^2+^-dependent high affinity binding to rat C2 at neutral pH but lacking pH-dependent target release.

### 3.2 Single-dose pharmacokinetics of the anti-rat C2 antibody in male Sprague Dawley rats

**Figure 3:**
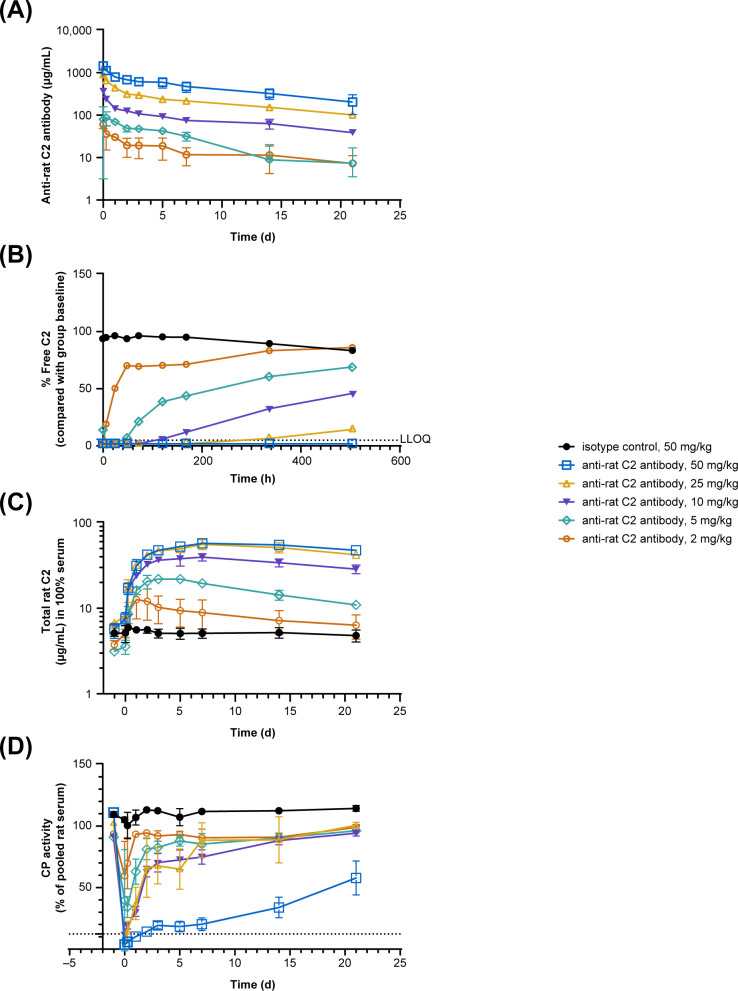
Single-dose pharmacokinetic and pharmacodynamic profiles of the anti-rat C2 antibody in male Sprague Dawley rats. Groups of three rats received a dose of anti-rat C2 antibody or an isotype control antibody as indicated. **(A)** Time course of serum anti-rat C2 antibody concentration. Data are mean and SD per group. **(B)** Percentage serum free C2 levels compared to average of baseline values of each group. Note that free C2 in the highest dose group E is reduced by ≥95% (= below Lower Limit of Quantification) for up to 21 days. **(C)** Total serum C2 levels. Data are as mean and SD per group. **(D)** Course of classical pathway activity measured as C3 deposition in ELISA (see methods) and expressed as % of the activity of pooled rat serum. Dotted line represents MgEGTA control.

To evaluate the PK and PD characteristics of the anti-rat C2 antibody, single ascending doses of the mAb were injected IV in eight-week-old male Sprague Dawley rats. Serum concentrations of the antibody were measured at multiple time points and used to estimate PK parameters using a non-compartmental model (**Supplementary Table 1B**). The C_max_ was anticipated at the initial bleeding time point following the intravenous injection. Overall, anti-rat C2 antibody exposure (C_max_, AUC) increased in a dose-proportional manner over the 2–50 mg/kg dose range tested (**Figure 3A**). The apparent terminal half-life was estimated at ~12.5 days, however the PK study duration was insufficient to accurately determine this parameter.

### 3.3 Pharmacodynamics of the anti-rat C2 antibody in male Sprague Dawley rats

In addition to the PK, different PD read outs were assessed to capture the characteristics of the anti-rat C2 antibody *in vivo*. The time course of free C2 (i.e., C2 unbound to mAb) and total C2 values in serum with their fitted curves after a single IV dose in male Sprague Dawley rats are depicted in **Figures 3B, C**, respectively. A dose-dependent reduction of free C2 was observed for the different anti-rat C2 antibody-treated groups (**Figure 3B**). The highest dose (50 mg/kg) resulted in reductions of ≥95% compared to isotype-treated animals for up to 21 days.

**Figure 3C** shows that total C2 levels dose-dependently increased in the rats upon administration of anti-rat C2 antibody. Saturation of C2 build-up was reached when dosing ≥25 mg/kg. Both the 25 mg/kg and 50 mg/kg dosed animals exhibited a 10-fold increase in total C2.

Next, the course of CP activity in serum was measured *in vitro* as another PD measure for complement blocking activity. Administration of the anti-rat C2 antibody reduced classical pathway activity in a dose-dependent manner (**Figure 3D**). Complete inhibition, which is a similar inhibition as that by MgEGTA, an established inhibitor of CP activity, was observed upon administration of 50 mg/kg mAb for up to 2 days. Complement activity gradually restored over time but had not reached baseline levels after 21 days. All other dose groups returned to their baseline CP activity values after 14 days or even earlier, depending on the dose.

### 3.4 Population PK/PD modeling and dose selection

A two-compartment model with linear elimination and zero-order infusion could adequately describe PK data. The PD component of the model captured the synthesis, degradation, and interaction with free C2 using a target binding model. A schematic representation of the final PK/PD model is presented in **Supplementary Figure 2A**. The final model included inter-individual variability (IIV) on all parameters and used a proportional residual error model. Model-based simulations revealed that a loading dose of 50 mg/kg followed by a maintenance dose of 25 mg/kg administered 3 days later, would be able to sustain reductions of free C2 concentrations above a threshold of ≥95% for at least 7 days (**Supplementary Figure 2B**).

### 3.5 *In vivo* efficacy of the anti-rat C2 antibody in a rat kidney transplantation model

**Figure 4:**
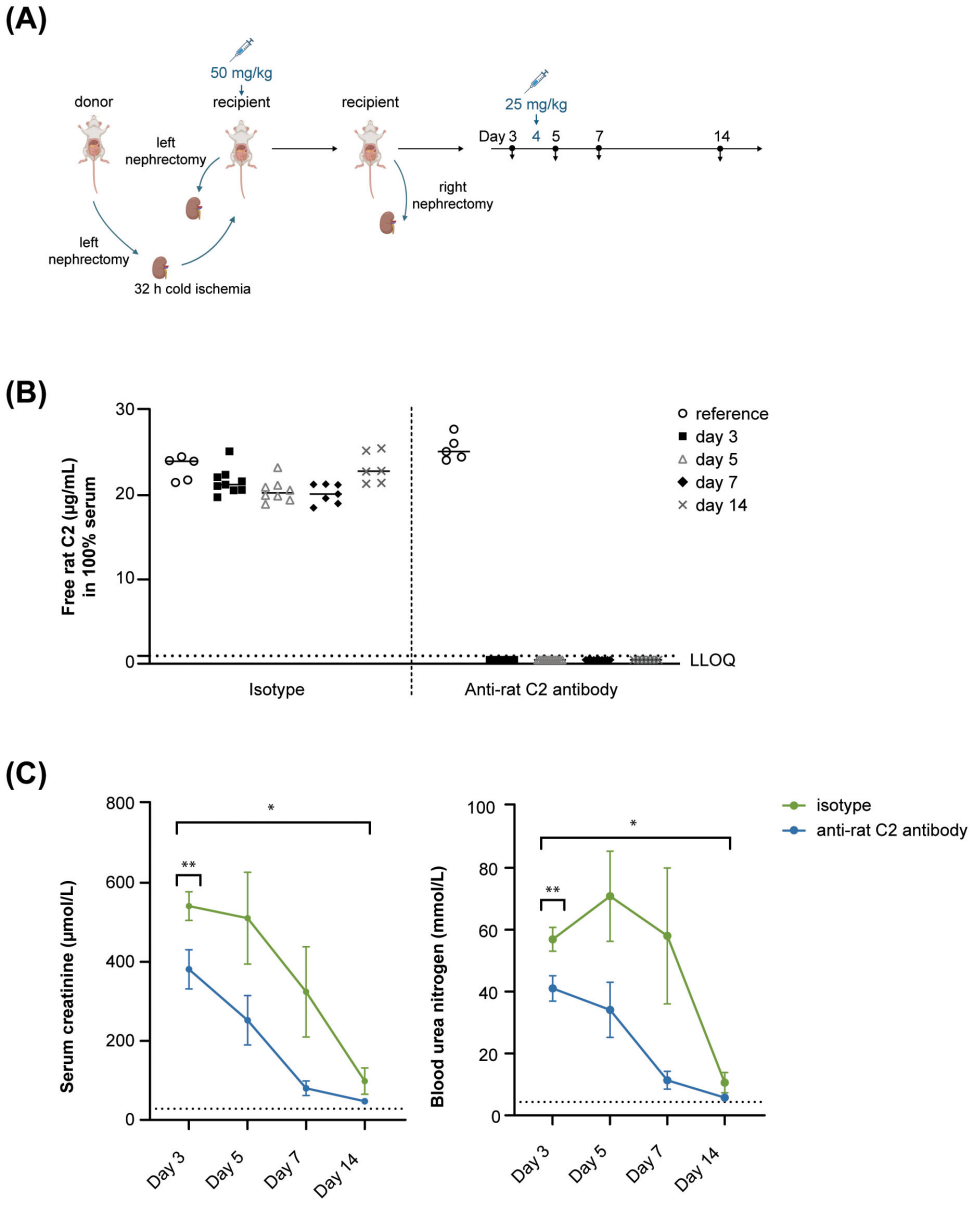
Anti-rat C2 antibody treatment during and post-kidney transplantation improves renal function. **(A)** Experimental study design. Recipient rats received a kidney transplant on day 1 that was previously exposed to 32 hours of cold ischemia. The contralateral kidney was then removed. Anti-rat C2 or an isotype control mAb was given IV just prior to reperfusion (day 1, 50 mg/kg), and 72 hours later (day 4, 25 mg/kg). Blood samples were collected on days 3, 5, 7, and 14. Transplanted kidneys were collected on day 14 for histological examination. **(B)** Free C2 serum levels in isotype (left) or anti-C2 (right) antibody-treated animals. LLOQ: 0.9375 µg/mL in 100% rat serum which corresponds to ≥95% reduction of free C2 as compared to reference animals. Pre-transplant donor serum values are added as reference. Anti-rat C2 antibody reduced free C2 levels for more than 95% for the full duration of the experiment. **(C)** Serum creatinine and blood urea nitrogen (BUN) concentrations. Mean pre-transplant concentrations in donor rats (n = 15) are shown as dotted lines. Day 3 creatinine and BUN analyses were repeated in an independent experiment with similar findings. *p < 0.05; **p < 0.01. IV, intravenous, LLOQ, Lower Level of Quantification. N = 18, 11, 9, and 7 for the anti-rat C2 mAb group and 9, 8, 7, and 6 for the isotype control mAb group on days 3, 5, 7, and 14, respectively.

The rat kidney transplantation model is summarized in **Figure 4A**. During the entire experiment, free C2 serum concentrations of anti-rat C2 antibody-treated rats were reduced by ≥95% as compared to reference levels in pre-surgery donor animals (**Figure 4B**). Rats receiving isotype control mAb developed severe kidney failure, as shown by a drastic increase in serum creatinine and BUN levels 2 days post-transplant (day 3, **Figure 4C**). Creatinine and BUN levels reduced over time post-transplant but were still higher on the day of sacrifice (day 14) compared to pre-transplant donor reference values. In contrast, in anti-rat C2 antibody-treated rats, serum creatinine and BUN concentrations were significantly lower than in isotype control rats 2 days post-transplant and remained lower up to the day of sacrifice. Creatinine levels in anti-rat C2 antibody-treated rats were on average 40% lower on day 3 (95% CI [14%; 59%], *p* = 0.008) and 49% lower across time points (95% CI [15%; 70%], *p* = 0.014). Additionally, these animals also had significantly reduced BUN concentrations at day 3 (33%, 95% CI [13%; 49%], *p* = 0.005, **Figure 4C**) and across time points (49%, 95% CI [13%; 70%], *p* = 0.017) compared to isotype-treated animals. Interestingly, NGAL release in serum was lower in rats treated with the anti-rat C2 mAb, suggesting that the improved renal clearance was related to less severe acute kidney injury (**Supplementary Figure 3**).

Notably, three rats in each treatment group died. All these mortality cases were associated with high serum creatinine post-transplantation, but only two deaths per treatment arm were considered related to IRI, based on microscopic findings, associated secondary changes in distant organs, and renal impairment (data not shown). The other two deaths were considered to be related to the transplantation/surgery procedure. Mortality rates were too low for meaningful conclusions. As planned sacrifice and unplanned mortality/sacrifice numbers were similar across treatment groups, missing values due to death were considered randomly distributed and were not taken into account in statistical analyses of creatinine, BUN, and NGAL levels. Furthermore, treatment did not impact incidence or severity of post-operative clinical signs, body weights, or body weight gains (data not shown).

Transplanted kidneys from all surviving animals were histologically examined on day 14, at which time the acute injury is expected to have subsided and changes are more reflective of (sub)chronic changes. Indeed, in isotype-treated control rats, IRI had resulted in gross and microscopic findings consistent with regeneration and subacute/chronic inflammation (**Table 1**). Specifically, kidneys appeared enlarged, discolored, and with abnormal consistency. Microscopically, IRI was evidenced by findings of tubular regeneration, hyaline/granular casts (within necrotic and regenerating tubules in the medulla, cortex and/or papilla), mineralization (mineralized sloughed necrotic tubular epithelial cells), and infiltration of interstitial lymphocytes and plasma cells accompanied by fibroplasia (**Table 1**). No notable histological changes were observed in glomeruli. Importantly, these tubular IRI-related findings were less in severity and incidence in animals treated with anti-rat C2 antibody than in isotype-treated control rats (**Table 1**). This reduction of IRI-related histological findings correlated with a decrease of the kidney weights of 23% as compared to the isotype-treated control group (**Table 1**), thereby suggesting a protective effect of complement blockade on kidney health at 2 weeks post-transplant.

**Table 1:** IRI-related histological findings in transplanted kidneys on day 14.

Group	Isotype control	anti-rat C2 antibody
Number of transplanted kidneys examined	6	7
Mean transplanted kidney weight at day 14		
Absolute weight (g)	2.66 ± 0.58	2.05 ± 0.40
% difference	NA	-22.91%
IRI-related gross pathology at day 14		
Enlargement	(2)	(0)
Discoloration, pale/dark/ mottled	(4)	(2)
Irregular surface	(0)	(0)
Abnormal consistency	(0)	(0)
IRI-related microscopic findings at day 14		
Regeneration; tubular	(6)	(7)
Minimal	0	0
Mild	0	3
Moderate	2	2
Marked	2	2
Severe	2	0
Inflammation, mononuclear cell	(6)	(7)
Minimal	0	4
Mild	6	3
Casts, hyaline/granular	(5)	(7)
Minimal	1	5
Mild	4	2
Mineralization	(6)	(2)
Minimal	3	2
Mild	2	0
Moderate	1	0

Six and seven kidneys were studied of isotype control and anti-rat C2 antibody-treated rats, respectively, due to unscheduled sacrifices/deaths of some rats. Percent weight difference is compared to the mean weight of isotype control kidneys on day 14. Numbers in parentheses represent the number of animals with the finding. IRI, ischemia-reperfusion injury.

A select number of animals were examined by histology at earlier time points (**Supplementary Table 2**). Although sample sizes were too small to assess treatment response during the peak of acute injury, histological assessment of these cohorts confirmed that the chronic inflammatory and regenerative responses, observed at day 14, were preceded by (sub)acute inflammation and tubular cell injury. Specifically, tubular necrosis, characterized by injured, markedly attenuated or hypereosinophilic epithelia, was observed in all four animals that prematurely died from IRI and in 3 out of 7 animals from the scheduled cohorts. Furthermore, interstitial and luminal neutrophil infiltration was observed in 5 animals, confirming activation of innate immunity.

## 4 Discussion

The ability of an antibody against a human protein to bind to homologous epitopes of various animal species facilitates the translation of interventional studies in animal models to human disease. Conversely, the absence of such cross-reactivity complicates clinical development of a therapeutic antibody. Empasiprubart binds to and blocks human C2 in a calcium- and pH-dependent fashion, which endows this mAb with recycling properties resulting in a unique PK/PD profile, but it has a low affinity for rat C2 (9). To facilitate preclinical evaluation of this antibody, we developed a high affinity homologue against rat C2 using the sequences and structure of empasiprubart and which, therefore, strongly resembles the functional characteristics of empasiprubart. The antibody potently blocks rat CP and LP activity *in vitro.* Moreover, just as empasiprubart, the antibody binds to rat C2 in a calcium-dependent manner, illustrated by the increase and decrease in maximum MSD signal at neutral pH when calcium or EDTA were added to the binding buffer, respectively. However, unlike empasiprubart, the anti-rat C2 antibody did not show pH-dependent dissociation from rat C2 as shown by the remaining rat C2 binding maximum MSD signal upon washing with a low pH buffer. Interestingly, this monoclonal antibody also cross reacted with human, cynomolgus monkey and hamster C2.

The PK/PD properties of our anti-rat C2 antibody were determined in male Sprague Dawley rats. A hIgG1 backbone format was selected, as this IgG subclass is more efficiently recycled than rat IgG1 via rat FcRn which should reflect prolonged PK (17). The PK/PD study revealed that the highest dose of anti-rat C2 antibody (50 mg/kg) led to free C2 reductions of ≥ 95% throughout the entire 21-day study period. Despite this long-term reduction in free C2, CP activity was suppressed for only 2 days at this dose; presumably this reflects that C2 levels are not a bottle-neck in the *in vitro* test as it uses high-density IgG antibodies (18, 19). Interestingly, total C2 levels in the rats increased upon administration of anti-C2 antibody, which is similar to the effect of empasiprubart in humans and further supports the resemblance of the rat anti-C2 antibody to empasiprubart. We postulate that this increase reflects inhibition of a spontaneous basal activation of CP and LP by the anti-rat C2 antibody, and/or its ability to prolong the circulating half-life of C2 through preventing clearance by unknown protein receptors.

The complement system plays a crucial role in several inflammatory diseases and conditions, including DGF after kidney transplantation. Overall, 40% of deceased donor kidney transplants have poor immediate renal clearance function, which necessitates dialysis in the first week post-transplant, a condition defined as DGF (20). Importantly, DGF is associated with worse long-term renal function and increased allograft loss (21–23). A major mechanism underlying DGF is ischemia reperfusion injury (IRI), which is more severe upon prolonged cold storage of the allograft. The pathophysiological process underlying IRI is complex and involves an intricate interplay between damaged or even dead cells, coagulation, complement, and inflammation. The pathophysiological role of complement is supported by experimental models and human data (24–27). Mechanisms may include cell lysis, interplay with the coagulation pathway by tissue factor induction and the generation of microparticles, and attraction and activation of neutrophils (28–31).

Involvement of specific complement pathways may be species-specific. In mice, for instance, inhibition of factor B or genetic deficiency of this factor reduces renal injury upon IR (32, 33), whereas a C4-deficiency does not provide protection (33, 34), suggesting that the AP plays a dominant role in renal IRI in mice. In contrast, non-human primates treated with CP/LP C1-inhibitor at the time of kidney transplant, had a lower DGF risk, superior urine output, and faster creatinine decline (7). This C1-inhibitor has also been studied in a phase 1/2 placebo-controlled, investigator-initiated, human clinical trial, where it was used as part of induction therapy. Although the incidence of DGF as defined by strict criteria was not reduced, C1-inhibitor reduced the need for dialysis 2 to 4 weeks post-transplant, improved estimated glomerular filtration rate at 1 year, and reduced graft loss at 3 years (35, 36).

In rats, several renal IRI models have been described that may partially translate into clinical DGF (11, 37). To support its potential as a tool to assess complement involvement in preclinical rat models for disease, the anti-rat C2 antibody was evaluated in a rat kidney transplant model (11). The rat model uses syngeneic donor organs, thereby preventing acute rejection risk and omitting the need for background immunosuppressive therapy. Previously, the complement inhibitor TT30 was shown to improve recipient creatinine recovery and survival in this model when administered to the ischemic organ, but in contrast to a C5-inhibitor, it had little effect when administered to the recipient during the reperfusion period (11). The beneficial effects of C2 blockade on kidney function and histological findings in our study were comparable to those observed upon inhibition of C5 (data not shown). Thus, the classical and/or lectin complement pathway seems to be (a) major contributor(s) to IR-induced kidney injury, at least in rats.

Post-transplant mortality due to IRI in our study was low which was unexpected given the dependency of the recipients on an organ exposed to a very long cold ischemia time, and the lack of any immunosuppressive therapy. Furthermore, creatinine levels more or less recovered at two weeks post-transplantation. A short ischemic period during transplantation can have major consequences on the long-term, such as development of interstitial fibrosis and tubular atrophy (38). In our study, the severity of tubular regeneration and inflammation two weeks post-transplant were improved in the rats treated with anti-rat C2 antibody. Thus, the anti-rat C2 antibody reduced tissue damage at early time points following IR in the rats and/or improved the healing process with faster resolution of inflammation, and restoration of normal tubular morphology, supporting a role for complement in IRI-related kidney damage in this model. Aberrant regeneration post-IRI can drive progression from acute to chronic kidney injury (39). So reduced inflammation upon intervention with anti-C2 treatment may contribute to improved long-term outcome of kidney transplantation (39). Future studies should reveal the validity of this concept, particularly in the clinic.

In conclusion, we have successfully generated a high affinity, calcium-dependent anti-C2 monoclonal antibody, an analogue of empasiprubart, that potently inhibits CP and LP of rats and effectively improves renal outcome in a rat kidney transplant model, underscoring its potential in various rat disease models. The observed favorable effects in the rat kidney transplant model provide preclinical support for the growing clinical interest in C2-targeted complement inhibition and for the ongoing clinical trial VARVARA (NCT05907096), evaluating empasiprubart in kidney transplant patients at risk for DGF (40).

## Data Availability

The raw datasets supporting the conclusions in this article are not readily available but can be requested by qualified researchers who engage in rigorous independent scientific research and can be provided after review and approval of a research proposal and statistical analysis plan, and execution of a data sharing agreement. Data requests can be submitted at any time and the data will be accessible for 12 months. argenx is committed to responsible data sharing regarding the clinical trials and research they fund. Requests to access the datasets should be directed to ESR@argenx.com.

## Supplementary material

Supplementary Figure 1 **(A)** The effect of empasiprubart and anti-rat C2 antibody on complement activation via the LP. Pooled rat serum was pre-incubated in the presence or absence of antibody or in the presence of 10 mM MgEGTA and added to the coated mannan and C3 deposition assayed. **(B)** The effect of empasiprubart and anti-rat C2 antibody on complement activation via the AP. Pooled rat serum was pre-incubated in the presence or absence of antibody and in the presence of 10 mM MgEGTA and added to coated LPS. **(C)** Binding kinetic profiles at neutral pH of a dilution series (0.52 nM–33.33 nM) of empasiprubart or anti-rat C2 antibody to either recombinant human or recombinant rat C2 analyzed by SPR. LP, lectin pathway; SPR, surface plasmon resonance.

Supplementary Figure 2 **(A)** Pharmacokinetic-pharmacodynamic model of anti-rat C2 antibody and free C2 dynamics. V1: volume of central compartment (L), V2: volume of peripheral compartment (L), CL: clearance from central compartment (L/h), Q: intercompartmental clearance (L/h), Ksyn; Free C2 synthesis rate constant (mg/h), Kdeg: Free C2 degradation rate constant (1/h), Kon: association rate constant (1/h), Koff: dissociation rate constant (1/h). **(B)** Result of the final PK/PD model simulation using the selected dosing regimen showing the free C2 levels below LLOQ (95.99% free C2 reduction) up to 168 h. h, hour; LLOQ, Lower Limit of Quantification. PD, pharmacodynamic; PK, pharmacokinetic.

Supplementary Figure 3 Anti-rat C2 antibody treatment during and post-kidney transplantation reduced post-transplant serum NGAL levels. For study set-up, refer to **Figure 4A**. Average pre-transplant donor serum value was 123.0 +- 20.9 ng/mL (mean +- SD). Serum was analyzed using the commercial Rat Lipocalin-2 ELISA Kit (ab119602) according to manufacturer’s protocol. Data was analyzed as fold change compared to pooled serum levels from healthy Lewis rats (79.7 +- 6.6 ng/mL; mean +- SD). **p* = 0.016 on day 7 and *p* = 0.062 on day 5. N = 17, 11, 9, and 7 for the anti-rat C2 mAb group and 9, 8, 7, and 6 for the isotype control mAb group on days 3, 5, 7, and 14 respectively. One animal of the anti-C2 mAb group was excluded (day 3) due to technical issue during analysis.

Supplementary Table 1 **(A)** Wash and dilution buffers of Meso Scale Discovery experiments. **(B)** Summary of average PK parameters for anti-rat C2 hIgG1 LALA antibody after single IV administration of 2, 5, 10, 30, and 50 mg/kg in male Sprague Dawley rats. hIgG1, human immunoglobulin G1; IV, intravenous; PK, pharmacokinetic.

Supplementary Table 2 IRI-related histological findings in transplanted kidneys on days 3-8. Kidneys were examined from rats sacrificed at prospectively scheduled time points (3 at day 3, and 2 at days 5 and 7) and from 4 rats which died prematurely (or were sacrificed for reaching humane endpoints) due to kidney IRI. Numbers in parentheses represent the number of animals with the finding. IRI, ischemia-reperfusion injury. d, days.
