# Supplementary material for: Development of an anti-rat complement C2 antibody that improves renal outcome in a rat kidney transplant model
Source: Front Immunol. 2026 Jan 15;16:1668376. doi: 10.3389/fimmu.2025.1668376 (PMC12852032; doi:10.3389/fimmu.2025.1668376)
Supplement: Supplementary Figure 1 — (A) The effect of empasiprubart and anti-rat C2 antibody on complement activation via the LP. Pooled rat serum was pre-incubated in the presence or absence of antibody or in the presence of 10 mM MgEGTA and added to the coated mannan and C3 deposition assayed. (B) The effect of empasiprubart and anti-rat C2 antibody on complement activation via the AP. Pooled rat serum was pre-incubated in the presence or absence of antibody and in the presence of 10 mM MgEGTA and added to coated LPS. (C) Binding kinetic profiles at neutral pH of a dilution series (0.52 nM–33.33 nM) of empasiprubart or anti-rat C2 antibody to either recombinant human or recombinant rat C2 analyzed by SPR. LP, lectin pathway; SPR, surface plasmon resonance. [file Supplementaryfile1.docx]

**Supplementary Information for**

**Development of an anti-rat complement C2 antibody that improves renal outcome in a rat kidney transplant model**

Laura Bracke^1†^, Jolien Delaere^1†^, Eline Haspeslagh^1^, Karen De Winter^1^, Yasmine Driege^1^, Raphael Bilgraer^1^, Tim Delahaye^1^, C. Erik Hack^1^, Inge Van de Walle^1*^

^†^*These authors contributed equally to this work*

^1^argenx, Industriepark 7, B-9052 Zwijnaarde, Belgium

*Corresponding author: ivandewalle@argenx.com

**SUPPLEMENTARY FIGURE 1**

**(A)** The effect of empasiprubart and anti-rat C2 antibody on complement activation via the LP. Pooled rat serum was pre-incubated in the presence or absence of antibody and in the presence of 10 mM MgEGTA and added to the coated mannan and C3 deposition assayed. **(B)** The effect of empasiprubart and anti-rat C2 antibody on complement activation via the AP. Pooled rat serum was pre-incubated in the presence or absence of antibody or in the presence of 10 mM MgEGTA and added to coated LPS. **(C)** Binding kinetic profiles at neutral pH of a dilution series (0.52 nM–33.33 nM) of empasiprubart or anti-rat C2 antibody to either recombinant human or recombinant rat C2 analyzed by SPR, LP, lectin pathway; SPR, surface plasmon resonance.


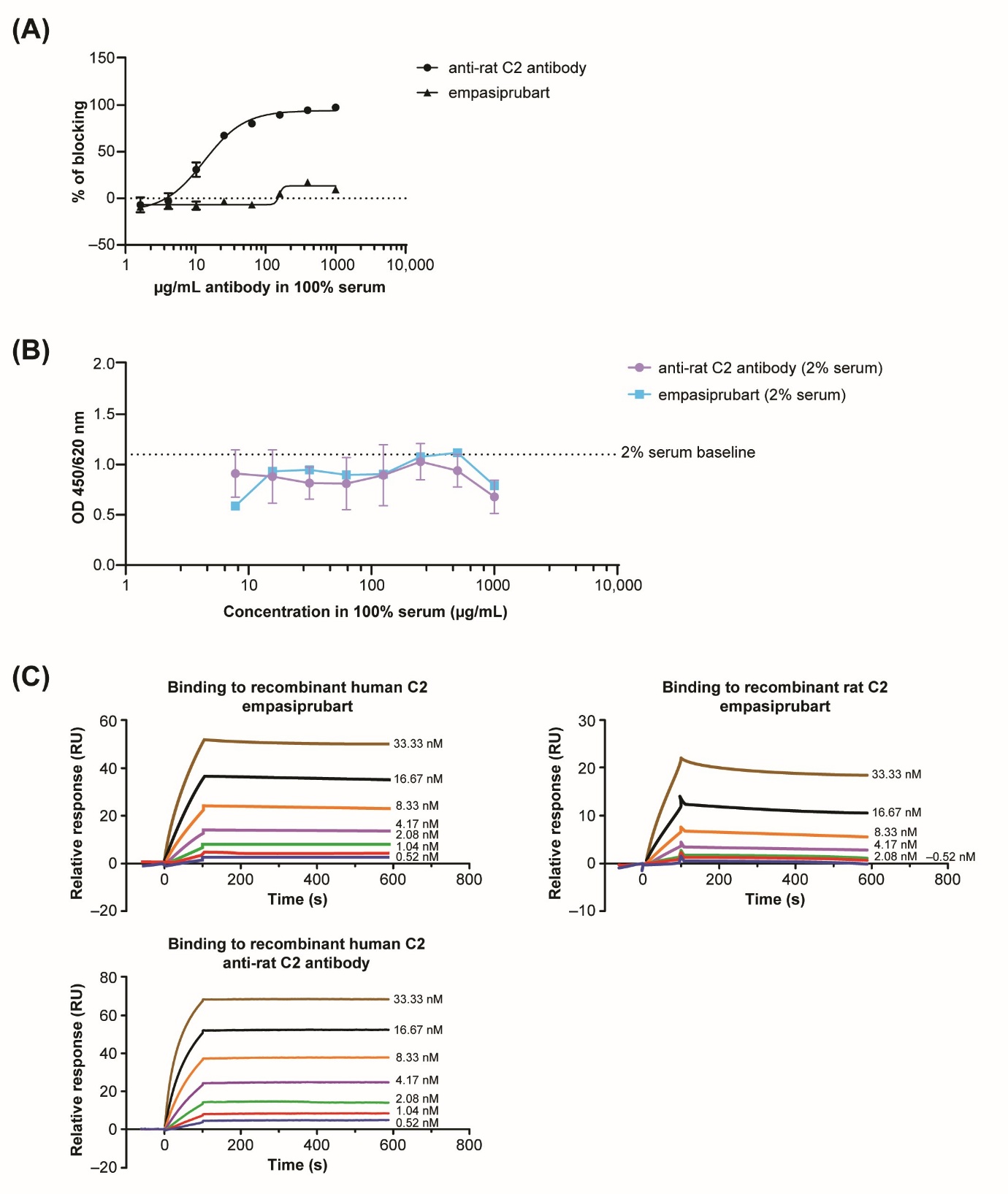


**SUPPLEMENTARY FIGURE 2**

**
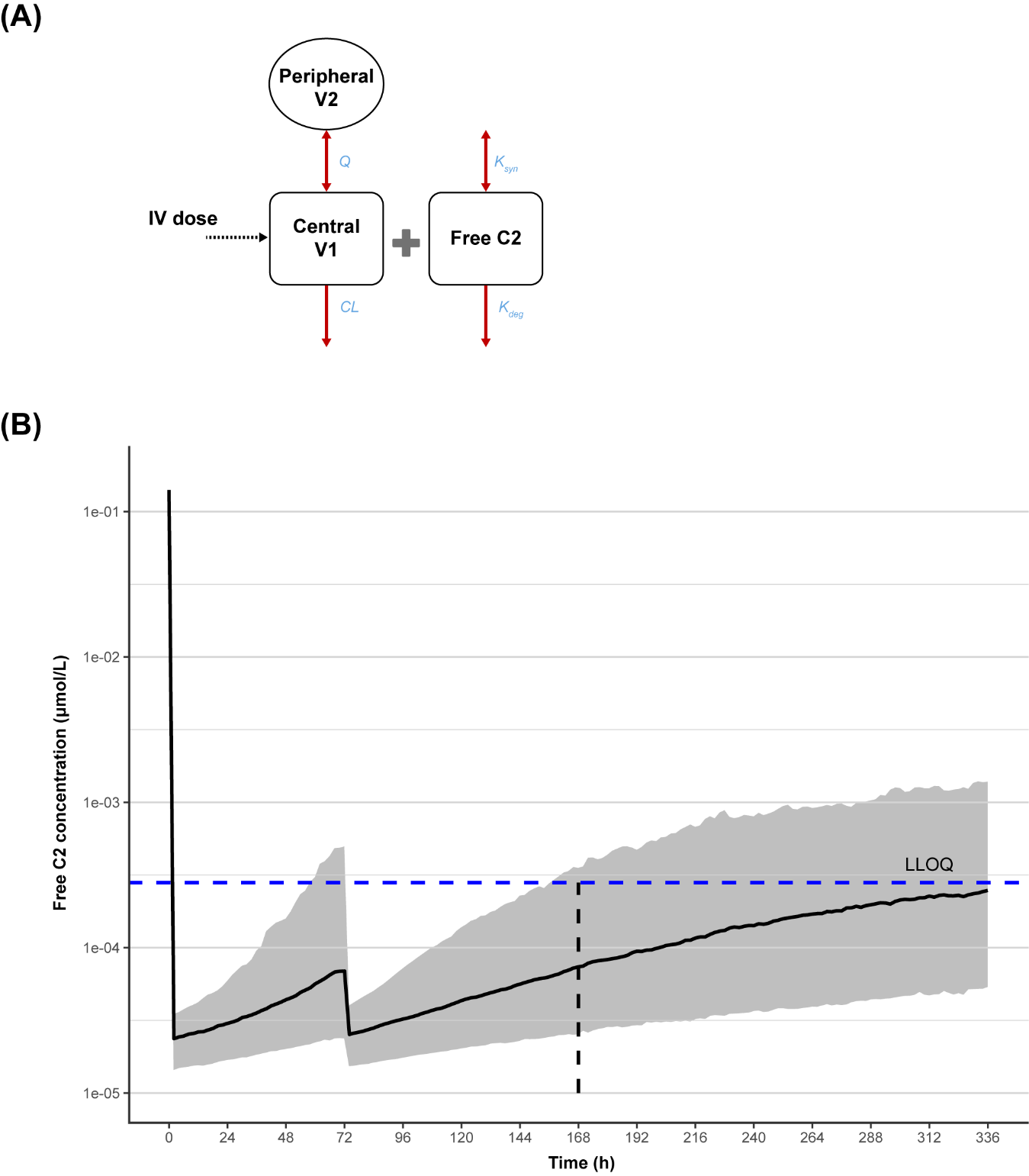
**

**(A)** Pharmacokinetic-Pharmacodynamic model of anti-rat C2 antibody and free C2 dynamics. V1: volume of central compartment (L), V2: volume of peripheral compartment (L), CL: clearance from central compartment (L/h), Q: intercompartmental clearance (L/h), Ksyn; Free C2 synthesis rate constant (mg/h), Kdeg: Free C2 degradation rate constant (1/h), Kon: association rate constant (1/h), Koff: dissociation rate constant (1/h). **(B)** Result of the final PK/PD model simulation using the selected dosing regimen showing the free C2 levels below LLOQ (95.99% free C2 reduction) up to 168 h. h, hour; LLOQ, Lower Limit of Quantification; PD, pharmacodynamic; PK, pharmacokinetic.

**SUPPLEMENTARY FIGURE 3**

Anti-rat C2 antibody treatment during and post-kidney transplantation reduced post-transplant serum NGAL levels. For study set-up, refer to Figure 4A. Average pre-transplant donor serum value was 123.0 +- 20.9 ng/mL (mean +- SD). Serum was analysed using the commercial Rat Lipocalin-2 ELISA Kit (ab119602) according to manufacturer’s protocol. Data was analysed as fold change compared to pooled serum levels from healthy Lewis rats (79.7 +- 6.6 ng/mL; mean +- SD). **p*=0.016 on Day 7 and *p*= 0.062 on day 5. N = 17, 11, 9 and 7 for the anti-rat C2 mAb group and 9, 8, 7 and 6 for the isotype control mAb group on days 3, 5, 7 and 14 respectively. One animal of the anti-C2 mAb group was excluded (day 3) due to technical issue during analysis.


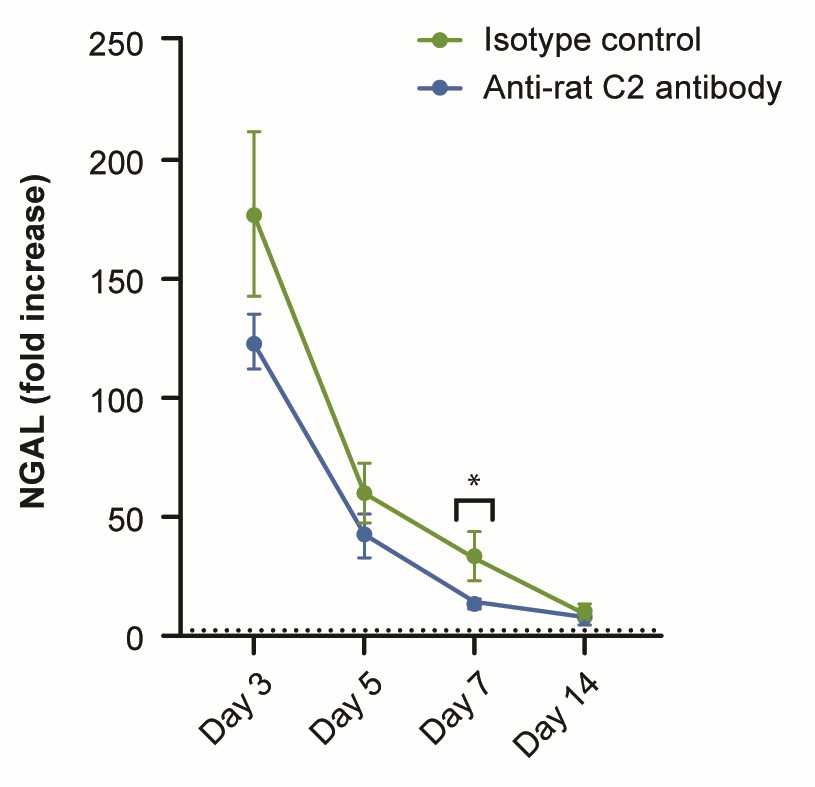


**SUPPLEMENTARY TABLE 1**

**A**

|  | **Wash buffer** | **Dilution buffer** |
| --- | --- | --- |
| **A** | TBS-0.05% Tween20, pH 7.4 | TBS-0.05%, pH 7.4, 0.1% wt/vol BSA |
| **B** | TBS-0.05% Tween20, pH 7.4, 1.25 mM CaCl2 | TBS, pH 7.4, 1.25 mM CaCl2, 0.1% wt/vol BSA |
| **C** | TBS-0.05% Tween20, pH 7.4, 20 mM EDTA | TBS pH 7.4, 20 mM EDTA, 0.1% wt/vol BSA |
| **D** | Citrate buffer, pH 5.5 (0.2 mol/L citric acid, 0.2 mol/L sodium citrate, 150 mmol/L NaCl), 0.05% tween20 | Citrate buffer, pH 5.5 (0.2 mol/L citric acid, 0.2 mol/L sodium citrate, 150 mmol/L NaCl), 0.1% wt/vol BSA |

**B**

| **Parameters** | **Dose level (mg/kg) anti-rat C2 antibody** | | | | |
| --- | --- | --- | --- | --- | --- |
|  | 2^b^ | 5^b^ | 10 | 30 | 50 |
| **Cmax, µg/mL** | 50.8 | 109 | 368 | 907 | 1450 |
| **DN_C_max,_ µg/mL/mg** | 25.4 | 21.8 | 36.8 | 36.3 | 29.0 |
| **t_max,_ day** | 0.0417-1^c^ | 0.0417-1^c^ | 0.0417 | 0.0417 | 0.0417 |
| **AUC_last,_ day*µg/mL** | 289 | 516 | 1560 | 4430 | 9270 |
| **DN_AUC_last,_ day*µg/mL/mg** | 145 | 103 | 156 | 177 | 185 |
| **t_½_^a^, day** | [10.2] | [15.8] | [12.1] | [12.7] | [11.8] |

^a^Parameter did not meet the described acceptance criteria and should be interpreted with caution, values are presented between [ ]. ^b^A few animals were most likely dosed paravenous. ^c^Range

DN: Dose normalized

**(A)** Wash and dilution buffers of Meso Scale Discovery experiments. **(B)** Summary of average PK parameters for anti-rat C2 hIgG1 LALA antibody after single IV administration of 2, 5, 10, 30, and 50 mg/kg in male Sprague Dawley rats. hIgG1, human immunoglobulin G1; IV, intravenous; PK, pharmacokinetic.

**SUPPLEMENTARY TABLE 2**

|  | **d3** | **d4** | | **d5** | | | **d7** | **d8** |
| --- | --- | --- | --- | --- | --- | --- | --- | --- |
| **Group** | **Anti-rat C2 antibody** | | **Anti-rat C2 antibody** | | **Anti-rat C2 antibody** | **Isotype control** | **Anti-rat C2 antibody** | **Isotype control** |
| Type of death | Scheduled | | Unscheduled | | Scheduled | Unscheduled | Scheduled | Unscheduled |
| Number of transplanted kidneys examined | 3 | | 2 | | 2 | 1 | 2 | 1 |
| **IRI-related gross pathology**  Enlargement  Discoloration, pale/dark/mottled  Abnormal irregular surface | (1)  (3)  (0) | | (2)  (0)  (0) | | (2)  (2)  (0) | (1)  (1)  (0) | (2)  (1)  (1) | (0)  (0)  (0) |
| **IRI-related microscopic findings** |  | |  | |  |  |  |  |
| Necrosis, tubular  Minimal Mild | (2) 0 2 | | (2) 0 2 | | (1) 1 0 | (1) 0 1 | (0) 0 0 | (1) 0 1 |
| Cast, hyaline/granular  Minimal Mild Moderate Marked | (3) 0 0 0 3 | | (2) 0 0 2 0 | | (2) 0 0 1 1 | (1) 0 0 1 0 | (2) 1 1 0 0 | (1) 0 0 1 0 |
| Mineralization  Minimal Mild Marked | (2) 2 0 0 | | (2) 1 1 0 | | (2) 1 1 0 | (1) 0 1 0 | (1) 0 1 0 | (1) 0 0 1 |
| Regeneration, tubular  Moderate Marked | (3) 3 0 | | (2) 2 0 | | (2) 0 2 | (1) 1 0 | (2) 1 1 | (1) 1 0 |
| Inflammation, mixed cell  Minimal Mild Moderate | (0) 0 0 0 | | (1) 1 0 0 | | (2) 0 2 0 | (1) 1 0 0 | (0) 0 0 0 | (1) 0 0 1 |
| Inflammation, mononuclear cell  Minimal  Mild | (0)  0  0 | | (1)  1  0 | | (0)  0  0 | (0)  0  0 | (2)  1  1 | (0)  0  0 |
| Hemorrhage  Minimal  Mild | (0)  0  0 | | (1)  1  0 | | (1)  0  1 | (1)  0  1 | (0)  0  0 | (0)  0  0 |

IRI-related histological findings in transplanted kidneys on days 3-8. Kidneys were examined from rats sacrificed at prospectively scheduled time-points (3 at day 3, and 2 at days 5 and 7) and from 4 rats which died prematurely (or were sacrificed for reaching humane endpoints) due to kidney IRI.

Numbers in parentheses represent the number of animals with the finding. IRI, ischemia-reperfusion injury. d, days.
